# Supplementary material for: A computational model elucidates the effects of oncogene-induced expression alterations on the energy metabolism of neuroblastoma
Source: Sci Rep. 2025 Sep 24;15:32708. doi: 10.1038/s41598-025-18656-w (PMC12460609; doi:10.1038/s41598-025-18656-w)
Supplement: Supplementary file 1 — Supplementary Material 1 [file 41598_2025_18656_MOESM1_ESM.pdf]

Supplementary Material

A computational model elucidates the effects of oncogene-induced expression alterations on the energy metabolism of neuroblastoma

Mareike Simon, Uwe Benary, Katharina Baum, Alexander Schramm, Jana Wolf

Ordinary differential equations

$$\frac{\mathrm{d}[glc]}{\mathrm{d}t} = \mathrm{v}_{\mathrm{glc\_tp}} - \mathrm{v}_{\mathrm{HK}}$$

$$\frac{\mathrm{d}[glc6p]}{\mathrm{d}t} = \mathrm{v}_{\mathrm{HK}} - \mathrm{v}_{\mathrm{GPI}}$$

$$\frac{\mathrm{d}[fru6p]}{\mathrm{d}t} = \mathrm{v}_{\mathrm{GPI}} - \mathrm{v}_{\mathrm{PFK1}}$$

$$\frac{\mathrm{d}[fru16bp]}{\mathrm{d}t} = \mathrm{v}_{\mathrm{PFK1}} - \mathrm{v}_{\mathrm{ALD}}$$

$$\frac{\mathrm{d}[dhap]}{\mathrm{d}t} = \mathrm{v}_{\mathrm{ALD}} - \mathrm{v}_{\mathrm{TPI}}$$

$$\frac{\mathrm{d}[gap]}{\mathrm{d}t} = \mathrm{v}_{\mathrm{ALD}} + \mathrm{v}_{\mathrm{TPI}} - \mathrm{v}_{\mathrm{GAPDH}}$$

$$\frac{\mathrm{d}[bpg]}{\mathrm{d}t} = \mathrm{v}_{\mathrm{GAPDH}} - \mathrm{v}_{\mathrm{PGK}}$$

$$\frac{\mathrm{d}[3pg]}{\mathrm{d}t} = \mathrm{v}_{\mathrm{PGK}} - \mathrm{v}_{\mathrm{PGM}}$$

$$\frac{\mathrm{d}[2pg]}{\mathrm{d}t} = \mathrm{v}_{\mathrm{PGM}} - \mathrm{v}_{\mathrm{ENO}}$$

$$\frac{\mathrm{d}[pep]}{\mathrm{d}t} = \mathrm{v}_{\mathrm{ENO}} - \mathrm{v}_{\mathrm{PK}}$$

$$\frac{\mathrm{d}[pyr]}{\mathrm{d}t} = \mathrm{v}_{\mathrm{PK}} - \mathrm{v}_{\mathrm{LDH}} - \mathrm{v}_{\mathrm{PDH}}$$

$$\frac{\mathrm{d}[lac]}{\mathrm{d}t} = \mathrm{v}_{\mathrm{LDH}} - \mathrm{v}_{\mathrm{lac\_tp}}$$

$$\frac{\mathrm{d}[acoa]}{\mathrm{d}t} = \mathrm{v}_{\mathrm{PDH}} - \mathrm{v}_{\mathrm{TCA}}$$

$$\frac{\mathrm{d}[adp]}{\mathrm{d}t} = \mathrm{v}_{\mathrm{HK}} + \mathrm{v}_{\mathrm{PFK1}} - \mathrm{v}_{\mathrm{PGK}} - \mathrm{v}_{\mathrm{PK}} - \mathrm{v}_{\mathrm{TCA}} - 14 \cdot \mathrm{v}_{\mathrm{Resp}} + \mathrm{v}_{\mathrm{ATP\_cons}}$$

$$\frac{\mathrm{d}[atp]}{\mathrm{d}t} = -\mathrm{v}_{\mathrm{HK}} - \mathrm{v}_{\mathrm{PFK1}} + \mathrm{v}_{\mathrm{PGK}} + \mathrm{v}_{\mathrm{PK}} + \mathrm{v}_{\mathrm{TCA}} + 14 \cdot \mathrm{v}_{\mathrm{Resp}} - \mathrm{v}_{\mathrm{ATP\_cons}}$$

$$\frac{\mathrm{d}[nad]}{\mathrm{d}t} = -\mathrm{v}_{\mathrm{GAPDH}} + \mathrm{v}_{\mathrm{LDH}} - \mathrm{v}_{\mathrm{PDH}} - 4 \cdot \mathrm{v}_{\mathrm{TCA}} + 6 \cdot \mathrm{v}_{\mathrm{Resp}}$$

$$\frac{\mathrm{d}[nadh]}{\mathrm{d}t} = \mathrm{v}_{\mathrm{GAPDH}} - \mathrm{v}_{\mathrm{LDH}} + \mathrm{v}_{\mathrm{PDH}} + 4 \cdot \mathrm{v}_{\mathrm{TCA}} - 6 \cdot \mathrm{v}_{\mathrm{Resp}}$$

$$\frac{\mathrm{d}[p]}{\mathrm{d}t} = -\mathrm{v}_{\mathrm{GAPDH}} - \mathrm{v}_{\mathrm{TCA}} - 14 \cdot \mathrm{v}_{\mathrm{Resp}} + \mathrm{v}_{\mathrm{ATP\_cons}}$$

$$\frac{\mathrm{d}[o2]}{\mathrm{d}t} = -3 \cdot \mathrm{v}_{\mathrm{Resp}} + \mathrm{v}_{\mathrm{o2\_tp}}$$

Conservation relations

The model includes three conservation relations. These include those for ATP and ADP as well as NAD and NADH since these are only converted into each other but not de novo synthesized in this model. The third one represents a conservation of the total phosphate availability and includes phosphate, ATP and all glycolytic intermediates from Glc6P to PEP.

**Name**	**Included metabolites**
Total adenosine phosphates	atp+adp
Total redox metabolites	nad+nadh
Total phosphate	atp+p+glc6p+fru6p+2*fru16bp+gap+dhap+2*bpg+3pg+2pg+pep

Rate equations

Most reactions are described by reversible Michaelis-Menten type kinetics. Oxygen transport and ATP consumption are described by mass-action kinetics. The mitochondrial reactions (PDH, TCA cycle, respiration) are modeled as irreversible. The oxygen transport is described by mass action kinetics, since it is based on diffusion. Since the complex GAPDH reaction was already modeled in a reduced form in Marín-Hernández et al. (2011), we here use the same rate law. The lumped reactions for the TCA cycle and respiration are adapted from Shestov et al. (2014). The k_m,k_i, k_a and k_{eq} values are specific for each reaction. Additional subscripts indicating the reaction name were omitted in the rate laws for easier readability.
ATP and ADP are representing also other nucleotide phosphates (e.g. GTP and GDP) and NAD and NADH are representing all redox metabolites (e.g. FAD and FADH2).

Glucose transporter

$$v_{glc\_tp} = \frac{\frac{vmf_{glc\_tp}}{k_m} \cdot ([glc\_ext] - \frac{[glc]}{k_{eq}})}{1 + \frac{[glc\_ext]}{k_m} + \frac{[glc]}{k_m}}$$

Hexokinase

$$v_{HK} = \frac{\frac{vmf_{HK}}{k_{m,glc} \cdot k_{m,atp}} \cdot ([glc] \cdot [atp] - \frac{[glc6p] \cdot [adp]}{k_{eq}})}{1 + \frac{[glc]}{k_{m,glc}} + \frac{[atp]}{k_{m,atp}} + \frac{[glc] \cdot [atp]}{k_{m,glc} \cdot k_{m,atp}} + \frac{[glc6p]}{k_{m,glc6p}} + \frac{[adp]}{k_{m,adp}} + \frac{[glc6p] \cdot [adp]}{k_{m,glc6p} \cdot k_{m,adp}}} \cdot \frac{k_{i,glc6p}}{k_{i,glc6p} + [glc6p]}$$

Glucose phosphate isomerase

$$v_{GPI} = \frac{\frac{vmf_{GPI}}{k_{m,glc6p}} \cdot ([glc6p] - \frac{[fru6p]}{k_{eq}})}{1 + \frac{[glc6p]}{k_{m,glc6p}} + \frac{[fru6p]}{k_{m,fru6p}}}$$

Phosphofructokinase

$$v_{PFK1} = \frac{\frac{vmf_{PFK1}}{k_{m,fru6p} \cdot k_{m,atp}} \cdot ([fru6p] \cdot [atp] - \frac{[fru16bp] \cdot [adp]}{k_{eq}})}{1 + \frac{[fru6p]}{k_{m,fru6p}} + \frac{[atp]}{k_{m,atp}} + \frac{[fru6p] \cdot [atp]}{k_{m,fru6p} \cdot k_{m,atp}} + \frac{[fru16bp]}{k_{m,fru16bp}} + \frac{[adp]}{k_{m,adp}} + \frac{[fru16bp] \cdot [adp]}{k_{m,fru16bp} \cdot k_{m,adp}}} \cdot \frac{k_{i,atp}}{k_{i,atp} + [atp]}$$

Aldolase

$$v_{ALD} = \frac{\frac{vmf_{ALD}}{k_{m,fru16bp}} \cdot ([fru16p] - \frac{[dhap] \cdot [gap]}{k_{eq}})}{1 + \frac{[fru16bp]}{k_{m,fru16bp}} + \frac{[dhap]}{k_{m,dhap}} + \frac{[gap]}{k_{m,gap}} + \frac{[dhap] \cdot [gap]}{k_{m,dhap} \cdot k_{m,gap}}}$$

Triose-phosphate isomerase

$$v_{TPI} = \frac{\frac{vmf_{TPI}}{k_{m,dhap}} \cdot ([dhap] - \frac{[gap]}{k_{eq}})}{1 + \frac{[dhap]}{k_{m,dhap}} + \frac{[gap]}{k_{m,gap}}}$$

Glyceraldehyde 3-phosphate dehydrogenase

$$v_{GAPDH} = \frac{\frac{vmf_{GAPDH}}{k_{m,nad} \cdot k_{m,gap} \cdot k_{m,p}} \cdot ([nad] \cdot [gap] \cdot [p] - \frac{[nadh] \cdot [bpg]}{k_{eq}})}{1 + \frac{[nad]}{k_{m,nad}} + \frac{[nad] \cdot [gap]}{k_{m,nad} \cdot k_{m,gap}} + \frac{[nad] \cdot [gap] \cdot [p]}{k_{m,nad} \cdot k_{m,gap} \cdot k_{m,p}} + \frac{[nadh]}{k_{m,nadh}} + \frac{[nadh] \cdot [bpg]}{k_{m,nadh} \cdot k_{m,bpg}}}$$

Phosphoglycerate kinase

$$v_{PGK} = \frac{\frac{vmf_{PGK}}{k_{m,bpg} \cdot k_{m,adp}} \cdot ([bpg] \cdot [adp] - \frac{[3pg] \cdot [atp]}{k_{eq}})}{1 + \frac{[bpg]}{k_{m,bpg}} + \frac{[adp]}{k_{m,adp}} + \frac{[bpg] \cdot [adp]}{k_{m,bpg} \cdot k_{m,adp}} + \frac{[3pg]}{k_{m,3pg}} + \frac{[atp]}{k_{m,atp}} + \frac{[3pg] \cdot [atp]}{k_{m,3pg} \cdot k_{m,atp}}}$$

Phosphoglycerate mutase

$$v_{PGM} = \frac{\frac{vmf_{PGM}}{k_{m,3pg}} \cdot ([3pg] - \frac{[2pg]}{k_{eq}})}{1 + \frac{[3pg]}{k_{m,3pg}} + \frac{[2pg]}{k_{m,2pg}}}$$

Enolase

$$v_{ENO} = \frac{\frac{vmf_{ENO}}{k_{m,2pg}} \cdot ([2pg] - \frac{[pep]}{k_{eq}})}{1 + \frac{[2pg]}{k_{m,2pg}} + \frac{[pep]}{k_{m,pep}}}$$

Pyruvate kinase

$$v_{PK} = \frac{\frac{vmf_{PK}}{k_{m,pep} \cdot k_{m,adp}} \cdot ([pep] \cdot [adp] - \frac{[pyr] \cdot [atp]}{k_{eq}})}{1 + \frac{[pep]}{k_{m,pep}} + \frac{[adp]}{k_{m,adp}} + \frac{[pep] \cdot [adp]}{k_{m,pep} \cdot k_{m,adp}} + \frac{[pyr]}{k_{m,pyr}} + \frac{[atp]}{k_{m,atp}} + \frac{[pyr] \cdot [atp]}{k_{m,pyr} \cdot k_{m,atp}}} \cdot (1 + \frac{[fru16bp]}{k_{a,fru16p}})$$

Lactate dehydrogenase

$$v_{LDH} = \frac{\frac{vmf_{LDH}}{k_{m,pyr} \cdot k_{m,nadh}} \cdot ([pyr] \cdot [nadh] - \frac{[lac] \cdot [nad]}{k_{eq}})}{1 + \frac{[pyr]}{k_{m,pyr}} + \frac{[nadh]}{k_{m,nadh}} + \frac{[pyr] \cdot [nadh]}{k_{m,pyr} \cdot k_{m,nadh}} + \frac{[lac]}{k_{m,lac}} + \frac{[nad]}{k_{m,nad}} + \frac{[lac] \cdot [nad]}{k_{m,lac} \cdot k_{m,nad}}}$$

Lactate transporter

$$v_{lac\_tp} = \frac{\frac{vmf_{lac\_tp}}{k_m} \cdot ([lac] - \frac{[lac\_ext]}{k_{eq}})}{1 + \frac{[lac]}{k_m} + \frac{[lac\_ext]}{k_m}}$$

Pyruvate dehydrogenase

$$v_{PDH} = \frac{vmax_{PDH} \cdot [pyr] \cdot [nad]}{k_{m,pyr} \cdot k_{m,nad} + [pyr] \cdot k_{m,nad} + [nad] \cdot k_{m,pyr} + [pyr] \cdot [nad]}$$

TCA cycle $$v_{TCA} = \frac{vmax_{CC} \cdot \frac{[acoa] \cdot [nad] \cdot [adp] \cdot [p]}{k_{m,acoa} \cdot k_{m,nad} \cdot k_{m,adp} \cdot k_{m,p}}}{(1 + \frac{[acoa]}{k_{m,acoa}}) \cdot (1 + \frac{[nad]}{k_{m,nad}}) \cdot (1 + \frac{[adp]}{k_{m,adp}}) \cdot (1 + \frac{[p]}{k_{m,p}})}$$

Respiration

$$v_{Resp} = \frac{vmax_{Resp} \cdot \frac{[o2] \cdot [nadh] \cdot [adp] \cdot [p]}{k_{m,o2} \cdot k_{m,nadh} \cdot k_{m,adp} \cdot k_{m,p}}}{(1 + \frac{[o2]}{k_{m,o2}}) \cdot (1 + \frac{[nadh]}{k_{m,nadh}}) \cdot (1 + \frac{[adp]}{k_{m,adp}}) \cdot (1 + \frac{[p]}{k_{m,p}})}$$

O_2 transport

$$v_{o2\_tp} = k1_{o2\_tp} \cdot ([o2_{ext}] - [o2])$$

ATP consumption

$$v_{ATP\_cons} = k1_{atp\_cons} \cdot [atp]$$

Parameters

The over 80 kinetic parameters including Michaelis-Menten constants and maximal velocities should be assigned in a neuroblastoma specific way. Where available experimentally determined values for neuroblastoma cells were used. km values were either estimated for neuroblastoma cells or are taken from the BRENDA (Jeske et al., 2019) and SABIO-RK (Wittig et al., 2012) databases. Here data for *Homo sapiens* were used whenever possible. Equilibrium constants are taken from literature (Holzhütter, 2004). The initial range of these values was set based on typical values for mammalian cells (as given in Mulukutla et al., 2014; Roy and Finley, 2017; Shestov et al., 2014), The maximal velocities, which are commonly assumed to be cell type specific, were then fitted using metabolomic data (for glc, g6p, fru16bp, dhap, 3pg, pep, pyr, lac, acoa, adp, nad, nadh) and the extracellular flux data (ECAR and OCR values from three independent experiments), each for three different glucose concentrations in the medium (5.55, 11.11 and 25 mM), from a neuroblastoma cell line as described in (Tjaden et al., 2020) (see Methods).

Table S.1: **List of parameters of the reference model.** The parameters are sorted by type and then alphabetically. The sources are: I for values from Holzhütter, 2004, II for parameters experimentally determined for this cell line (Tjaden et al., 2020), III for fitted parameters, IV for literature/database derived parameters (Jeske et al., 2019, Wittig et al., 2012). All concentrations, Michaelis Menten constants, inhibition and activation constants are given in mM, maximal velocities in mM/min. The units of the equilibrium constants depend on the number of products and substrates, for same number of substrates and products there is no unit. The table continues on the next page.

Parameter	Value	Source	Parameter	Value	Source
keq_ALD	0.114	I	k1_atp_cons	1.11	III
keq_ENO	1.7	I	k1_o2tp	10.28	III
keq_GAPDH	1.92E-04	I	vmax_CC	3.79	III
keq_glctp	1	I	vmax_PDH	1.89	III
keq_GPI	0.3922	I	vmax_resp	3.48	III
keq_HK	3.90E+03	I	vmf_ALD	5474.59	III
keq_lactp	1	I	vmf_ENO	5906.76	III
keq_LDH	9.09E+03	I	vmf_GAPDH	10000.00	III
keq_PFK1	1.00E+05	I	vmf_GPI	9998.71	III
keq_PGK	1460	I	vmf_HK	4.89	III
keq_PGM	0.1449	I	vmf_LDH	9999.81	III
keq_PK	1.38E+04	I	vmf_PFK1	4.98	III
keq_TPI	4.07E-02	I	vmf_PGK	9999.41	III
			vmf_PGM	8057.65	III
glc_ext	11.11	II	vmf_PK	23.00	III
lac_ext	0.5	I	vmf_TPI	8665.75	III
o2_ext	0.181	I	vmf_glctp	1.79	III
			vmf_lactp	10000.00	III

Parameter	Value	Source
km_ald_dhap	0.095	IV
km_ald_f16bp	0.0107	IV
km_ald_gap	1.17	IV
km_cc_acoa	0.278	III
km_cc_adp	3.599	III
km_cc_nad	0.071	III
km_cc_p_i	0.289	III
km_eno_2pg	0.03	IV
km_eno_pep	0.702	IV
km_gapdh_bpg	0.01	IV
km_gapdh_gap	0.149	IV
km_gapdh_nad	0.071	IV
km_gapdh_nadh	0.01	IV
km_gapdh_p	4	IV
Km_glctp	1.5	IV
km_gpi_f6p	0.0635	IV
km_gpi_g6p	0.445	IV
km_hk_adp	11.1	IV
km_hk_atp	0.46	II
km_hk_g6p	2	IV
km_hk_glc	0.064	II
ki_hk_g6p	0.24	IV
km_lactp	4.68	IV
km_ldh_lac	0.94	IV
km_ldh_nad	0.311	IV
km_ldh_nadh	0.014	IV
km_ldh_pyr	0.08	II
km_pdh_nad	0.033	IV
km_pdh_pyr	0.03	IV
km_pfk_adp	0.49	IV
km_pfk_atp	0.05	IV
km_pfk_f16bp	16.7	IV
km_pfk_f6p	0.45	IV
ki_pfk_atp	1.6	IV

Parameter	Value	Source
km_pgk_3pg	0.009	IV
km_pgk_adp	0.2	IV
km_pgk_atp	0.07	IV
km_pgk_bpg	0.024	IV
km_pgm_2pg	0.041	IV
km_pgm_3pg	0.4	IV
km_pk_adp	0.17	IV
km_pk_atp	0.35	IV
km_pk_pep	1.1	IV
km_pk_pyr	0.48	IV
ka_pk_f16bp	0.1	IV
km_resp_adp	0.001	III
km_resp_nadh	0.001	III
km_resp_o2	0.001	III
km_resp_p_i	10	III
km_tpi_dhap	0.17	IV
km_tpi_gap	1.76	II

Supplemental Figures

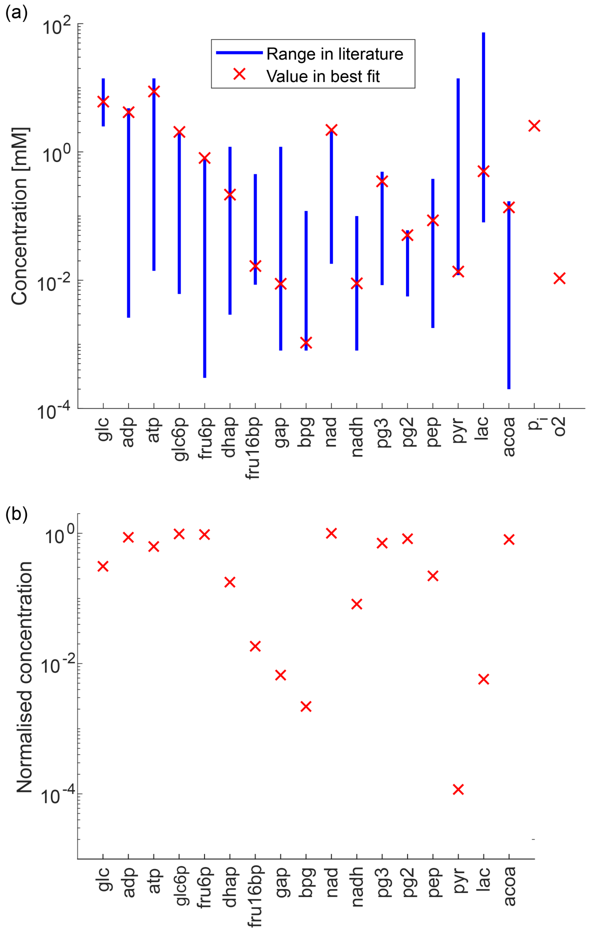

Figure S.1: **Comparison of fitted model concentrations to known literature values.** (a) Experimental range, shown by the blue bars, are taken from Roy and Finley (2017), these represent concentrations in different mammalian cell lines, mostly cancer cell lines. Therefore, they are not representative for a single cell type or neuroblastoma. Oxygen (o2) and phosphate (p_i) are not included in this experimental dataset. The red crosses represent the steady state concentrations of the model with the best parameter fit. (b) Visualisation of the concentrations of the best fit in their respective concentration range known in literature (Roy and Finley 2017) in a normalised form. The concentrations are normalised: Normalised Conc. $= (\text{Conc}_{\text{BestFit}} - \text{Min}_{\text{Lit.}})/(\text{Max}_{\text{Lit.}} - \text{Min}_{\text{Lit.}})$. Overall, the metabolite concentrations of the best fit correspond to the literature derived ranges, although some values are close to the boundaries.

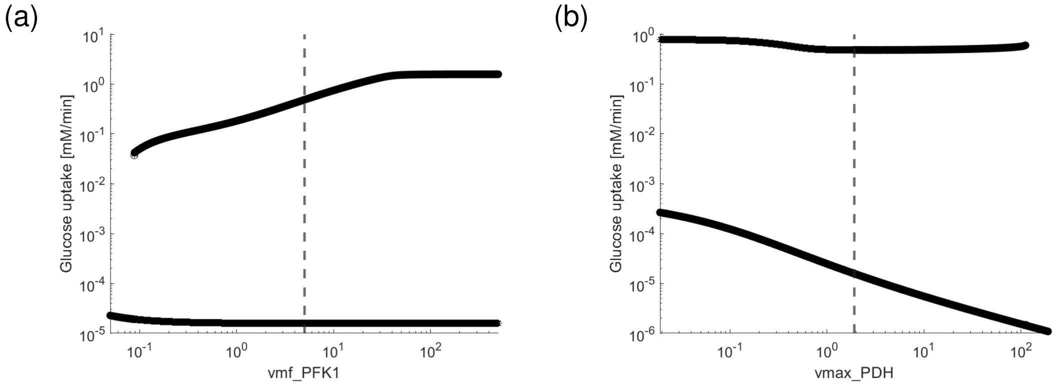

Figure S.2: **Coexistence of two stable steady states for additional parameters.** Stable steady state values in dependence of one parameter, for (a) maximal velocity of the PFK reaction and (b) the maximal velocity of the PDH reaction. The dashed line indicates the fitted value of the respective parameter. All other parameters are at their reference values (Supplemental Table S.1).

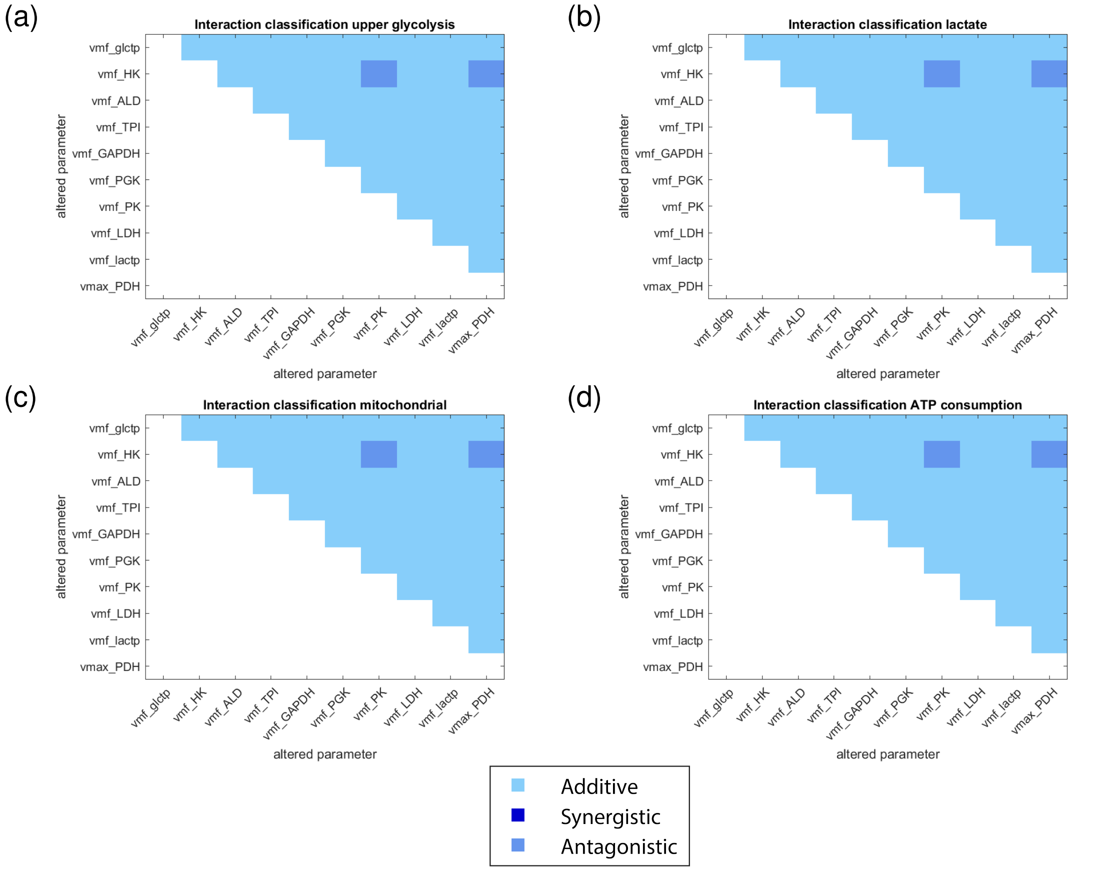

Figure S.3: **Interaction analysis of MYCN targets.** All MYCN targets are altered by a MYCN factor of two and the interaction effect between each possible pair of MYCN targets is analyzed. These effects are classified as either additive (light blue), synergistic (dark blue) or antagonistic (intermediate blue). While most effects are additive, two antagonistic cases can also be observed.

Supplemental Tables

Table S.2: **Steady state concentrations for the reference and low flux steady state.** All concentrations are given in mM.

Metabolite	Steady state concentration [mM] Reference state	Low flux state
glc	6.082	11.110
glc6p	2.052	2.134
fru6p	0.805	0.837
fru16bp	0.017	4.198
dhap	0.216	3.429
gap	0.009	0.140
bpg	0.001	7.70E-11
3pg	0.349	1.47E-07
2pg	0.050	2.11E-08
pep	0.086	3.56E-08
pyr	0.014	1.23E-06
lac	0.500	0.500
acoa	0.137	0.025
o2	0.011	0.181
atp	8.776	0.001
adp	4.168	12.943
nad	2.202	0.048
nadh	0.009	2.163
p	2.559	1.33E-04

Table S.3: **Steady state fluxes for different MYCN factors.**

	Steady state flux [mM/min]			
	Reference	MYCN factor		
Flux	state	1.1	1.5	2
glc uptake	0.48	0.50	0.56	0.62
lac release	0.38	0.42	0.57	0.75
o2 uptake	1.75	1.73	1.61	1.47
ATP consumption	9.71	9.62	9.17	8.57

Table S.4: **Steady state concentrations for different MYCN factors.**

	Steady state concentration [mM]		
	Reference	MYCN factor	
Metabolite	state	1.5	2
glc	6.082	7.045	7.615
glc6p	2.052	2.736	3.309
fru6p	0.805	1.073	1.298
fru16bp	0.017	0.003	0.001
dhap	0.216	0.085	0.038
gap	0.009	0.003	0.002
bpg	0.001	0.001	0.0004
3pg	0.349	0.282	0.222
2pg	0.050	0.041	0.032
pep	0.086	0.069	0.055
pyr	0.014	0.023	0.033
lac*	0.500	0.500	0.500
acoa	0.137	0.114	0.095
o2	0.011	0.024	0.038
atp	8.776	8.285	7.740
adp	4.168	4.659	5.204
nad	2.202	2.206	2.208
nadh	0.009	0.005	0.004
p	2.559	2.358	2.239

* Lactate concentrations are close to equilibrium, but the intracellular concentration is slightly higher than the extracellular concentration. This is not visible due to rounding effects.

Table S.5: **Leave-one-out-analysis for MYCN targets.** All MYCN targets except one are altered by a factor of 2. For comparison the top row shows the steady state fluxes for the case that all targets are altered, the last row shows the steady state if only five (glctp, HK, PGK, PK and PDH) of the targets are altered. Targets that lead to notable changes in the steady state fluxes are marked in grey.

Left out	glc_ tp [mM/min]	lac_ tp [mM/min]	o2_ tp [mM/min]	ATP_ cons [mM/min]
None	0.62	0.75	1.47	8.57
vmf_glctp	0.62	0.75	1.47	8.58
vmf_HK	0.49	0.40	1.71	9.52
vmf_ALD	0.62	0.75	1.47	8.57
vmf_TPI	0.62	0.75	1.47	8.57
vmf_GAPDH	0.62	0.75	1.47	8.57
vmf_PGK	0.62	0.75	1.46	8.56
vmf_PK	0.63	0.78	1.43	8.38
vmf_LDH	0.62	0.75	1.47	8.57
vmf_lactp	0.62	0.75	1.47	8.57
vmax_PDH	0.61	0.72	1.50	8.74
Simultaneous leave-out of 5 targets	0.62	0.75	1.47	8.57

Table S.6: **Steady state flux distribution among the best 10 fits.**

	glc_tp [mM/min]	lac_tp [mM/min]	o2_tp [mM/min]	ATP_cons [mM/min]
Best fit	0.481	0.379	1.750	9.714
2nd best fit	0.481	0.379	1.750	9.714
3rd best fit	0.481	0.379	1.750	9.714
4th best fit	0.481	0.379	1.749	9.707
5th best fit	0.483	0.382	1.751	9.719
6th best fit	0.482	0.381	1.745	9.687
7th best fit	0.483	0.381	1.753	9.731
8th best fit	0.483	0.381	1.753	9.731
9th best fit	0.483	0.381	1.753	9.731
10th best fit	0.483	0.381	1.753	9.730

Bibliography

Holzhütter, H. G. (2004). The principle of flux minimization and its application to estimate stationary fluxes in metabolic networks. *European journal of biochemistry / FEBS*, *271*(14), 2905–22.

Jeske, L., Placzek, S., Schomburg, I., Chang, A., & Schomburg, D. (2019). BRENDA in 2019: A European ELIXIR core data resource. *Nucleic Acids Research*, *47*(D1), D542–D549.

Marín-Hernández, A., Gallardo-Pérez, J. C., Rodríguez-Enríquez, S., Encalada, R., Moreno-Sánchez, R., & Saavedra, E. (2011). Modeling cancer glycolysis. *Biochimica et Biophysica Acta (BBA) - Bioenergetics*, *1807*(6), 755–767.

Mulukutla, B. C., Yongky, A., Daoutidis, P., & Hu, W. S. (2014). Bistability in glycolysis pathway as a physiological switch in energy metabolism. *PLoS ONE*, *9*(6).

Roy, M., & Finley, S. D. (2017). Computational Model Predicts the Effects of Targeting Cellular Metabolism in Pancreatic Cancer. *Frontiers in Physiology*, *8*(April), 1–16.

Shestov, A. A., Liu, X., Ser, Z., Cluntun, A. A., Hung, Y. P., Huang, L., Kim, D., Le, A., Yellen, G., Albeck, J. G., & Locasale, J. W. (2014). Quantitative determinants of aerobic glycolysis identify flux through the enzyme GAPDH as a limiting step. *eLife*, *3*, 1–18.

Tjaden, B., Baum, K., Marquardt, V., Simon, M., Trajkovic-Arsic, M., Kouril, T., Siebers, B., Lisec, J., Siveke, J. T., Schulte, J. H., Benary, U., Remke, M., Wolf, J., & Schramm, A. (2020). N-Myc-induced metabolic rewiring creates novel therapeutic vulnerabilities in neuroblastoma. *Scientific Reports*, *10*(1), 1–10.

Wittig, U., Kania, R., Golebiewski, M., Rey, M., Shi, L., Jong, L., Algaa, E., Weidemann, A., Sauer-Danzwith, H., Mir, S., Krebs, O., Bittkowski, M., Wetsch, E., Rojas, I., & Müller, W. (2012). SABIO-RK - Database for biochemical reaction kinetics. *Nucleic Acids Research*, *40*(D1), D790–D796.
